# Cervical Stump Cancer Treated With Radiotherapy Using Computed Tomography-Guided Brachytherapy

**DOI:** 10.7759/cureus.13789

**Published:** 2021-03-09

**Authors:** Kohei Okada, Takahiro Oike, Ken Ando, Nobuteru Kubo, Tatsuya Ohno

**Affiliations:** 1 Radiation Oncology, Gunma University Graduate School of Medicine, Maebashi, JPN; 2 Heavy Ion Medical Center, Gunma University, Maebashi, JPN; 3 Radiation Oncology, Gunma University Graduate School of Medicine, Maebash, JPN

**Keywords:** cervical stump cancer, radiation therapy, brachytherapy, computed tomography, rectosigmoid colon

## Abstract

Cervical stump cancer, which arises in the remaining uterine cervix of a woman with a history of supravaginal hysterectomy, accounts for 1.6-4.4% of all cervical cancers. The close proximity of the rectosigmoid colon to the primary tumor, which is due to the absence of the uterine corpus, should be considered carefully in the treatment planning of brachytherapy. Although three-dimensional image-guided brachytherapy (3D-IGBT) is used widely to treat cervical cancer in those with an intact uterine corpus, the safety and efficacy of 3D-IGBT for cervical stump cancer remains unclear. Here, we report a case of cervical stump cancer (T3bN1M0) treated successfully with definitive radiotherapy, which combined external beam radiotherapy and computed tomography (CT)-based IGBT. By applying the dose prescription concept used for definitive brachytherapy of cervical cancer with an intact uterine corpus, IGBT achieved satisfactory dose conformity to the tumor while sparing the adjacent rectosigmoid colon. This led to local tumor control for three years and eight months, with no late adverse effects. This case suggests that radiotherapy using CT-based IGBT is a safe and effective treatment for cervical stump cancer.

## Introduction

Cervical stump cancer occurs in the remaining uterine cervix of a woman who has undergone supravaginal hysterectomy for benign diseases such as uterine fibroids, benign ovarian tumors, and postpartum hemorrhage. Cervical stump cancer accounts for 1.6-4.4% of all cervical cancer cases [1-6]. Radiotherapy comprising external body radiotherapy (EBRT) and brachytherapy is the definitive treatment [7]. In patients with cervical stump cancer, the rectosigmoid colon is located in close proximity to the primary tumor due to the absence of the uterine corpus. Therefore, these patients are at higher risk of radiation exposure to the rectosigmoid colon during brachytherapy compared to patients with an intact uterus [8].

Historically, the dose prescription in brachytherapy for cervical cancer has been based on point A, i.e., the point 2 cm cranial and lateral to the external uterine ostium, to which 6 Gy is delivered per session. In recent years, three-dimensional image-guided brachytherapy (3D-IGBT) using magnetic resonance imaging (MRI) or computed tomography (CT) has become widespread, which enabled delivery of a sufficient dose to the tumor while sparing normal tissues [9, 10]. However, the safety and efficacy of 3D-IGBT for cervical stamp cancer remains unclear. Here, we report a case of cervical stump cancer treated successfully with definitive radiotherapy using CT-based IGBT.

## Case presentation

**Figure 1:**
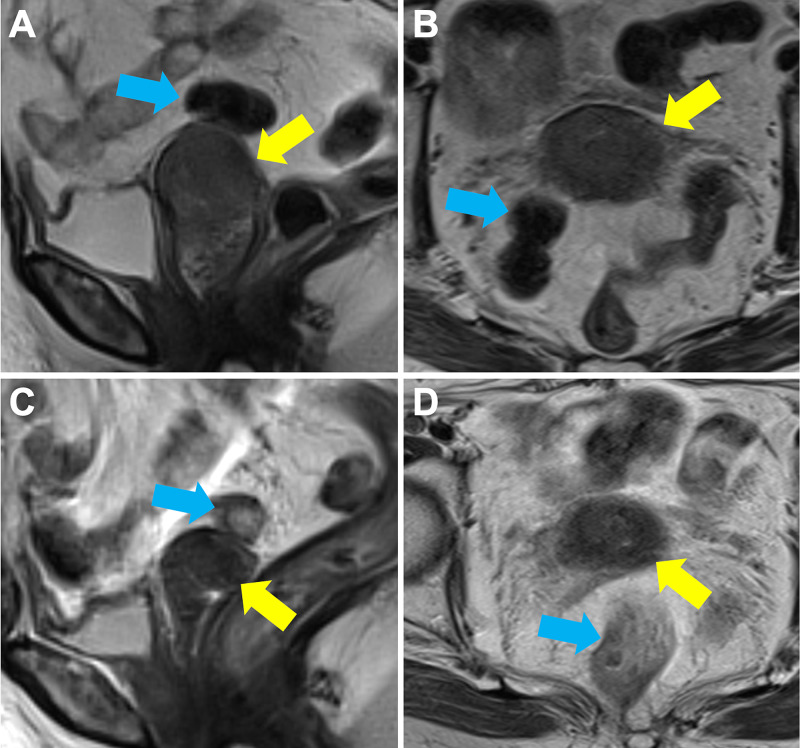
T2-weighted magnetic resonance images of the cervical stump tumor. (A) Time of diagnosis, sagittal plane. (B) Time of diagnosis, axial plane. (C) Before initiation of brachytherapy, sagittal plane. (D) Before initiation of brachytherapy, axial plane. Yellow arrows indicate the primary tumor. Blue arrows indicate the rectosigmoid colon located in close proximity to the tumor.

A 77-year-old woman consulted the Department of Gynecology and Obstetrics because of genital bleeding. The patient underwent supravaginal hysterectomy for the uterine fibroid at the age of 33 years. Bimanual examination identified a tumor in the remaining part of the uterine cervix. The tumor was pathologically diagnosed as squamous cell carcinoma. MRI revealed that the tumor was 42 × 33 × 32 mm in size, with bilateral parametrial involvement reaching the left pelvic wall (Figure 1A, 1B). 18F-fluorodeoxyglucose positron emission tomography showed lymph node metastasis to the left external iliac regions. Cystoscopy and colonoscopy revealed no tumor invasion of the bladder or rectum, respectively. Based on these findings, the disease was staged as T3bN1M0 according to the classification of Union for International Cancer Control (7th edition). Radiotherapy was selected as the definitive treatment due to the disease advancement and the patient was referred to the Department of Radiation Oncology. Chemotherapy was not considered due to her advanced age.

Radiotherapy comprised external beam radiotherapy (EBRT) and CT-based IGBT [11]. EBRT was performed at 2 Gy per fraction, five fractions per week. Whole pelvic irradiation was delivered at a total of 50 Gy; the latter 20 Gy was delivered using central shielding (3 cm-width) to spare the rectum and bladder. The superior border of the central shielding was set at the lower edge of the sacroiliac joint to cover the common iliac lymph node regions. In addition, a 6 Gy-boost was administered to the metastatic pelvic lymph nodes.

**Figure 2:**
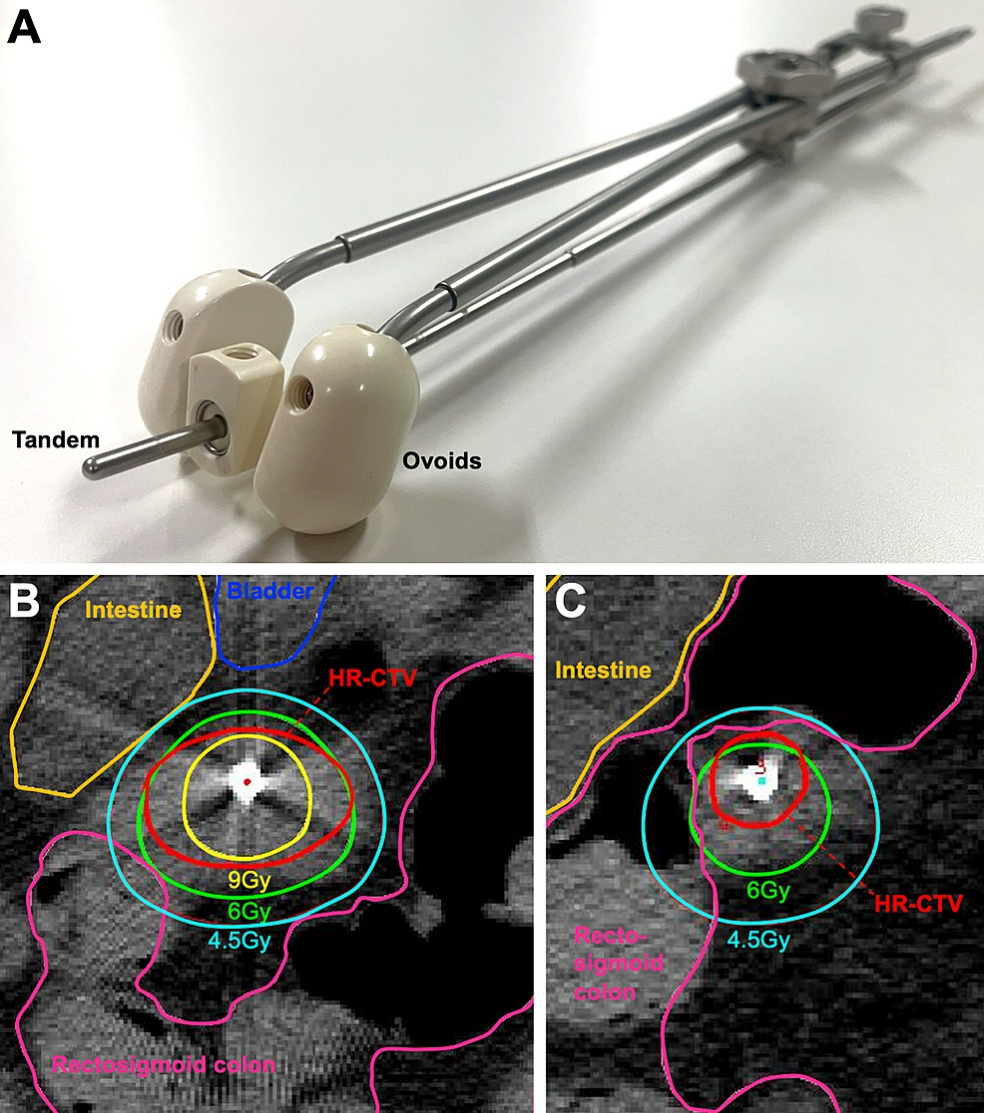
Brachytherapy settings. (A) The tandem (2 cm in depth) and ovoid applicators. (B, C) Representative images of computed tomography-based brachytherapy planning. (B) Axial plane in which the high risk-clinical target volume (HR-CTV, red) shows the maximal diameter. (C) Axial plane showing the cranial end of the HR-CTV. Note that the contours of HR-CTV are broadly covered by the iso-dose lines denoting 6 Gy (green), whereas those for the rectosigmoid colon (magenta) surrounding HR-CTV are not.

**Figure 3:**
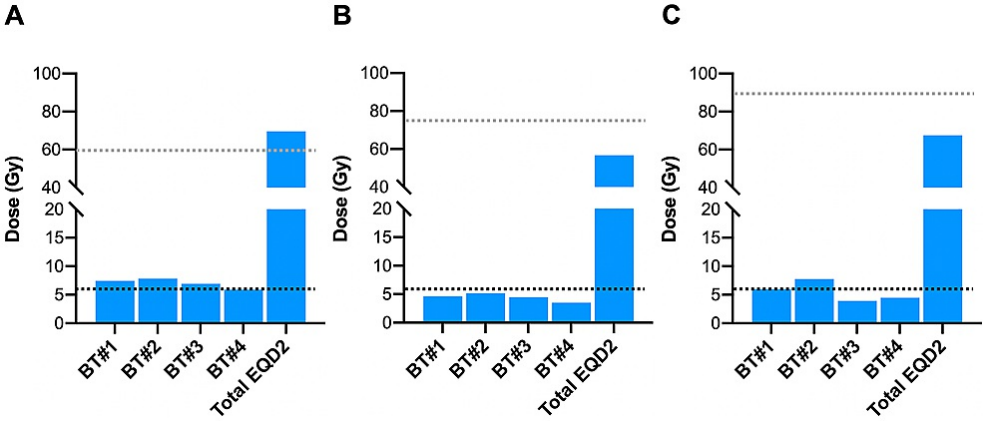
Dose-volume parameters used for computed tomography-based image-guided brachytherapy. (A) D90 (i.e., the minimum dose delivered to a given 90% of the target volume) of the high risk-clinical target volume (HR-CTV). (B) D2cc (i.e., maximum dose delivered to a given 2 cc of the target volume) of the rectosigmoid colon. (C) D2cc of the bladder. BT#1, #2, #3, and #4 indicate each brachytherapy session, respectively. The total equivalent dose in 2 Gy-fractions for external beam radiotherapy plus brachytherapy (EQD2) was calculated using an a/b ratio of 10 and 3 for the HR-CTV and for the organs at risk, respectively. Black dashed lines denote 6 Gy. Gray dashed lines (A), (B), and (C) denote 60 Gy, 75 Gy, and 90 Gy, respectively.

CT-based IGBT was performed for a total of four sessions (one session per week) from the fourth week of EBRT. A high-dose rate 192Ir remote-after-loading system (microSelectron, Elekta, Stockholm, Sweden) and tandem and ovoid applicators were used (N.B., the intrauterine cavity was conserved for approximately 2 cm in length) (Figure 2A). On the MRI performed before the first IGBT session, the primary tumor showed a fair response to EBRT (i.e., a reduction in size to 20 × 10 × 12 mm); however, the rectosigmoid colon was located in close proximity to the tumor (Figure 1C, 1D). The entire cervix and the surrounding tumor infiltrate were delineated as the high risk-clinical target volume (HR-CTV), and treatment plans were created with the goal of delivering 6 Gy or greater to the HR-CTV per session while minimizing the dose delivered to the rectosigmoid colon and bladder (Figure 2B, 2C) [11]. As a result, the average HR-CTV D90 (i.e., the minimum dose delivered to a given 90% of the target volume) over the four IGBT sessions was 7.0 Gy (range, 5.9-7.8 Gy). For the rectosigmoid colon, the average D2cc (i.e., the maximum dose delivered to a given 2 cc of the target volume) was 4.4 Gy (range, 3.5-5.1 Gy). For the bladder, the average D2cc was 5.5 Gy (range, 3.9-7.8 Gy). The total equivalent dose in 2 Gy-fractions for EBRT plus brachytherapy (EQD2) was calculated using an a/b ratio of 10 and 3 for HR-CTV and the organs at risk, respectively [12]. Thus, the total EQD2 for HR-CTV was 69.6 Gy, whereas that for the rectosigmoid colon and bladder was 56.7 Gy and 67.5 Gy, respectively (Figure 3).

The patient completed the planned treatment. Nausea, diarrhea, and urinary frequency (all assessed as Grade 1 according to the Common Terminology Criteria for Adverse Events, version 4) were noted as acute adverse effects. At three years and eight months after radiotherapy, the patient shows no evidence of recurrence or late adverse effects (e.g., the gastrointestinal or genitourinary hemorrhage, edema of the lower extremities, and pelvic bone fracture).

## Discussion

We report a case of cervical stump cancer treated successfully with radiotherapy. Treatment strategy for cervical stump cancer has not been standardized. Although early stage disease can be treated with surgical resection [13], our case had an advanced disease with bilateral parametrial involvement reaching the left pelvic wall that was considered to have no indication for this treatment. Therefore, we treated this case with radiotherapy following the clinical practice guideline for locally advance cervical cancer with an intact uterus [14]. Ideally, cisplatin-based chemotherapy should have been used concomitantly with radiotherapy as definitive treatment, this was not considered due to her advanced age. Nevertheless, our case achieved local tumor control without late adverse effects over three years and eight months, which may be worth noting.

CT-based IGBT achieved satisfactory dose sparing of the rectosigmoid colon located in close proximity to the tumor, thereby contributing to the absence of late adverse effects. This case indicates that radiotherapy using CT-based IGBT is a safe and effective treatment for cervical stump cancer.

Brachytherapy for patients with cervical stump cancer in the pre-IGBT era has resulted in a high incidence of gastrointestinal adverse effects. For example, Petersen et al. reported that 31% (14/46) of patients experienced serious adverse events in the rectum or sigmoid colon [5]. These data indicate the need to improve definitive treatment for cervical stump cancer.

CT-based IGBT is used widely to treat cervical cancer in those with an intact uterine corpus. For this subset of disease, the dose-response relationship for the rectum has been analyzed intensively. If we focus on studies that use central shielding techniques for EBRT (as in our case), Ohno et al. reported the outcome of definitive chemoradiotherapy using CT-based IGBT for 80 patients with locally advanced cervical cancer [11]. That study aimed to achieve a total EQD2 to the HR-CTV (D90) of >60 Gy and that to the rectum (D2cc) of <75 Gy. As a result, the five-year local control rate for stage III-IVA patients was as high as 90%, with no rectal toxicity greater than Grade 2 over a median follow-up of 60 months. Meanwhile, Okazaki et al. reported the outcome of definitive chemoradiotherapy using CT-based IGBT for 103 patients with locally advanced cervical cancer [15]. That study aimed to achieve a HR-CTV (D90) of >6 Gy at each brachytherapy session, with a total EQD2 to the rectum (D2cc) of <75 Gy. As a result, the two-year local control rate for stage III-IVA patients was 87%, and the incidence of rectal toxicity greater than Grade 2 was 2%. In our own case, the total EQD2 to HR-CTV (D90) was 69.6 Gy, and that to the rectosigmoid colon (D2cc) was 56.7 Gy, resulting in favorable local control over three years and eight months, with no late adverse effects. These data suggest that the standard dose constraints used when treating cervical cancer in those with an intact uterine corpus are feasible for CT-based IGBT for cervical stump cancer.

It should be discussed that radiotherapy following surgery may have greater risk for complications. The feasibility of definitive radiotherapy for cervical stump cancer warrants validation in prospective cohorts from this perspective, in which de-escalation of IGBT dose may be considered.

The limitation of this study is as follows. We made a diagnosis of bilateral parametrial involvement with the findings from bimanual examination, those from MRI, as well as the discussion with gynecologists taken into consideration. Nevertheless, it is difficult to exclude the possibility for these lesions being postoperative adhesion.

## Conclusions

Cervical stump cancer arises in the remaining uterine cervix of a woman with a history of supravaginal hysterectomy, and accounts for 1.6-4.4% of all cervical cancers. The close proximity of the rectosigmoid colon to the cervical stump tumor, due to the absence of the uterine corpus, should be considered carefully in the treatment planning of brachytherapy. In this case report, we showed that CT-based IGBT achieved sufficient dose sparing in the rectosigmoid colon located in close proximity to the tumor, leading to favorable local tumor control without late adverse effects. This case shows that radiotherapy using CT-based IGBT is a safe and effective treatment for cervical stump cancer. Validation in prospective studies is warranted.
